# Rhabdomyolysis Associated With Semaglutide Therapy: A Case Report

**DOI:** 10.7759/cureus.50227

**Published:** 2023-12-09

**Authors:** Sabrina A Billings, Heidi M Felix, Cara C Prier, Mary S Hedges

**Affiliations:** 1 Internal Medicine, Mayo Clinic, Jacksonville, Florida, USA

**Keywords:** drug-related side effects and adverse reactions, medication complications, myoglobinuria, obesity treatment, weight loss and obesity, glp1-ra, semaglutide, myalgias, rhabdomyolysis

## Abstract

This report describes the case of a 47-year-old woman with myalgias, weakness, and elevated creatine kinase associated with semaglutide therapy prescribed for weight loss. Her symptoms and laboratory markers were consistent with rhabdomyolysis and resolved after discontinuation of semaglutide. Upon rechallenge at a lower dose, symptoms recurred, and urinalysis was consistent with myoglobinuria. Symptoms again rapidly resolved upon discontinuation of the medication. It is imperative for physicians to recognize semaglutide as a possible cause of myalgias and rhabdomyolysis in clinically suspected patients. To the best of our knowledge, this is the first reported case in the literature and may be specific to semaglutide rather than a class effect of glucagon-like peptide 1 (GLP-1) agonists.

## Introduction

Rhabdomyolysis is characterized by clinical symptoms consisting of muscle pain, weakness, and tea-colored urine. Not all patients will have symptoms, and up to 50% of patients can be asymptomatic [1]. Regardless of the underlying causes, the global mortality rate for rhabdomyolysis is as high as 8% [1]. Given that this condition is caused by the breakdown of muscle cells and the release of their intracellular components, creatine kinase (CK) levels are expected to rise to five times the upper limit of normal or higher [2]. Multiple drugs have been identified as a cause for rhabdomyolysis, however, Glucagon-like peptide 1 (GLP-1) agonists have not been previously associated with this condition in the literature.

Multiple studies on GLP-1 agonists have demonstrated efficacy in weight loss, and both liraglutide and semaglutide currently have Food and Drug Administration (FDA) approval in the United States for the medical indications of obesity or overweight in the presence of at least one weight-related comorbidity [3]. Although adverse events have been reported with GLP-1 agonists, the majority of events have been gastrointestinal side effects and a lower incidence has been noted with semaglutide compared to other GLP-1 agonists [4]. 

In partnership with Mayo Clinic Libraries, PubMed and Embase database searches were conducted in August 2023, with the search terms GLP-1 agonist and rhabdomyolysis, which identified zero reports. A second search of both databases for GLP-1 and CK, myalgias, muscle pain, or muscle atrophy was undertaken and also did not yield any reports. Both searches included the time range of the past five years and publication in English. To our knowledge, we present the first reported case of GLP-1-induced rhabdomyolysis.

## Case presentation

A 47-year-old female with a history of hypothyroidism, fibromuscular dysplasia, and cerebral aneurysm presented to the primary care clinic with a burning sensation in her skin, muscular pain involving her right shoulder and right flank, as well as diffuse weakness, fatigue, and difficulty getting out of bed. Her symptoms occurred after two doses of semaglutide therapy for the indication of weight loss. Additional symptoms included diarrhea, nausea, migraine, and chills without fever. She denied symptoms of dyspnea, urticaria, or angioedema. Her medications included daily aspirin, duloxetine, levothyroxine, omeprazole, semaglutide as well as lasmiditan and ondansetron as needed for migraines. Her family history included obesity, hypothyroidism, migraines, and arthritis. Prior to semaglutide initiation, she tolerated liraglutide 3 mg subcutaneously daily with no side effects for five months with a concordant decline in her body mass index (BMI) from 33.3 kg/m^2^ to 25.9 kg/m^2^. Due to insurance coverage considerations, her dosage was subsequently switched to an equivalent dose of semaglutide 1.7 mg administered subcutaneously weekly. 

On examination, her vital signs were within normal range. Skin exam did not reveal erythema, subcutaneous edema, or any skin lesions. Musculoskeletal exam was negative for joint effusions and her range of motion was noted to be intact. Neurological exam found upper or lower extremity strength intact with five out of five strength tests reflecting successful muscle activation against the examiner's full resistance. Both light and deep palpitation of her skin and musculature diffusely led to patient's reported discomfort. The rest of the physical examination was unremarkable.

Laboratory evaluation revealed a normal complete blood cell (CBC) with a hemoglobin of 12.9 (normal: 11.6-15.0 g/dL) and white blood cell count of 5.7 (normal: 3.4-9.6 x10^9^L), and a normal differential without evidence of lymphocytosis. She also had normal creatinine, electrolytes, C-reactive protein (CRP), and cyanocobalamin (B12) levels. The liver panel revealed a newly isolated elevation of aspartate aminotransferase (AST) level of 73 U/L (normal: 8-43 U/L) with normal alanine aminotransferase (ALT) and normal alkaline phosphatase. CK level was found to be elevated at 2,619 U/L (normal: 26-192 U/L). Initial microscopic urinalysis was reported negative aside from 3 red blood cells/high power field (hpf) (normal: 0-2/hpf). While her lipase level was slightly elevated at 93, it was not indicative of pancreatitis, and subsequent abdominal ultrasound and CT scans did not reveal any abnormalities (Table 1).

**Table 1:** Laboratory values at presentation

Serum Laboratory	Day 0	Day 1	Day 4	Day 5	Normal Range
Hemoglobin (g/dL)	11.7	12.9		12.7	11.6-15.0
Hematocrit (%)	34.5	38.1		37.7	35.5-44.9
Platelets (x10^9^/L)	219	235		218	157-371
White blood cell count (x10^9^/L)	4.4	5.7		4.8	3.4-9.6
Neutrophils	2.90x10^9^/L (65.9%)	4.02x10^9^/L (70.3%)			1.56-6.45x10^9^/L (50-75%)
Lymphocytes	0.98x10^9^/L (22.3%)	1.04x10^9^/L (18.2%)			0.95-3.07x10^9^/L (10-42%)
Eosinophils	0.10x10^9^/L (2.3%)	0.13x10^9^/L (2.3%)			0.03-0.48x10^9^/L (1-3%)
Sodium (mmol/L)	138		139	139	135-145
Potassium (mmol/L)	4.0		4.0	4.7	3.6-5.2
Bicarbonate (mmol/L)	23		24	24	22-29
Creatinine (mg/dL)	0.89		0.83	0.86	0.59-1.04
Blood urea nitrogen (mg/dL)	14		10	13	6-21
Estimated glomerular filtration rate (mL/min/BSA)	81		88	84	>60
Calcium (mg/dL)	9.1		9.2	9.6	8.6-10.0
Phosphorous (mg/dL)	3.6		3.0	3.4	2.5-4.5
Alanine aminotransferase (U/L)	19	28	28	27	7-45
Aspartate aminotransferase (U/L)	52	73	47	34	8-43
Alkaline Phosphatase (U/L)	41	47	42	49	35-104
Bilirubin total (mg/dL)	0.5	0.4	0.6	0.4	< 1.2
Bilirubin direct (mg/dL)	0.1	0.1	0.2	0.1	0.0-0.3
Glucose (mg/dL)	85			93	70-140
Ferritin (mcg/L)	12				11-307
Folate (mcg/L)	13.9				> 4
Vitamin B12 (ng/L)	301				180-914
Thyroid-stimulating hormone (mlU/L)	2.7				0.3-4.2
Lipase (U/L)	93				13-60
Creatine kinase (U/L)		2619	712	354	26-192
C-reactive protein (mg/dL)		<3.0			< 8.0
Lactate dehydrogenase (U/L)		197			122-222
Aldolase (U/L)		7.1			< 7.7
Haptoglobin (mg/dL)		117			30-200
Urinalysis					
Glucose		Negative			Negative
Ketones		Negative			Negative
Bilirubin		Negative			Negative
Protein		Negative			Negative
Leukocyte Esterase		Negative			Negative
Nitrite		Negative			Negative
Specific gravity		1.013			1.002-1.030
pH		7.5			5.0-8.0
Red blood cells (urine RBC)		3/hpf			0-2/hpf
White blood cells (urine RBC)		0/hpf			0-5/hpf
Urine pregnancy				Negative	Not applicable

**Figure 1:**
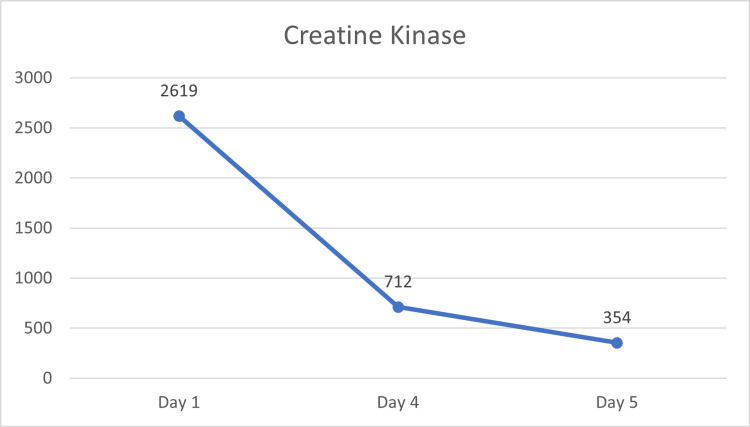
Laboratory serum creatine kinase upon initial evaluation and after semaglutide discontinuation

**Figure 2:**
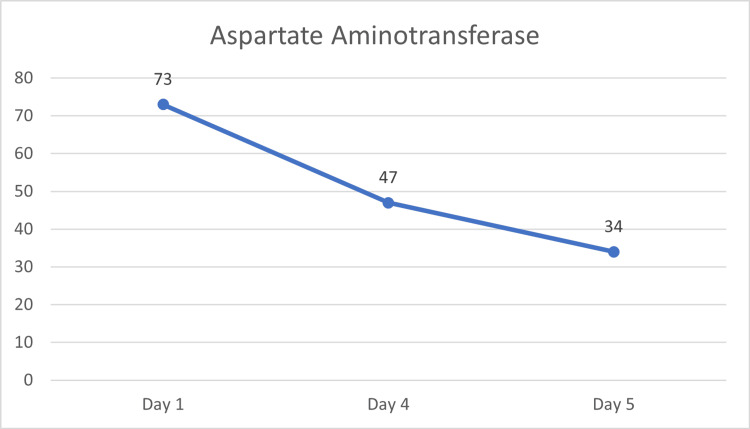
Laboratory serum aspartate aminotransferase upon initial evaluation and after semaglutide discontinuation

Given the suspicion for rhabdomyolysis and timing associated with semaglutide initiation, administration of the drug was discontinued. Her symptoms resolved and CK and AST levels normalized within days of discontinuation (Figure 1, Figure 2, Table 1).

Approximately four weeks later, the patient elected to re-trial semaglutide at a lower dose, starting at 0.5 mg with close monitoring while titrating upwards per recommended dosing. She initially tolerated the 0.5 mg dose and the 1 mg dose. However, at the 1.7 mg dose, her symptoms of myalgia, muscle weakness, and diffuse tenderness to palpitation recurred. Repeat serum CK was within the normal range at 51 U/L, but concomitant repeat microscopic urinalysis reported “moderate” hemoglobin (normal: negative) despite findings of zero red blood cells/hpf, suggesting myoglobinuria from damage to the cell membrane of myocytes. Expanded evaluation including autoimmune markers, and repeat liver function tests, electrolytes, creatinine, CRP, CBC with differential, and thiamine were within normal limits (Table 2). 

**Table 2:** Laboratory values upon retrial

Serum laboratory	Day 30 at dose 0.5 mg/0.5mL under the skin every 7 days	Day 14 of dose 1.7 mg/0.75 mL under the skin every 7 days, and recurrence of symptoms	Expanded laboratory evaluation 5 days after re-trial discontinuation	Normal range
Hemoglobin (g/dL)	12.9	13.1		11.6-15.0
Hematocrit (%)	39.3	39.3		35.5-44.9
Platelets (x10^9^/L)	228	240		157-371
White blood cell count (x10^9^/L)	5.0	6.1		3.4-9.6
Neutrophils		4.50x10^9^/L (74.5%)		1.56-6.45x10^9^/L (50-75%)
Lymphocytes		1x10^9^/L (16.5%)		0.95-3.07x10^9^/L (10-42%)
Eosinophils		0.11x10^9^/L (1.8%)		0.03-0.48x10^9^/L (1-3%)
Sodium (mmol/L)	137	140		135-145
Potassium (mmol/L)	4.5	4.6		3.6-5.2
Bicarbonate (mmol/L)	26	26		22-29
Creatinine (mg/dL)	0.90	0.90		0.59-1.04
Blood urea nitrogen (mg/dL)	19	17		6-21
Estimated glomerular filtration rate (mL/min/BSA)	80	80		>60
Calcium (mg/dL)	9.2	10.0		8.6-10
Phosphorous (mg/dL)	3.6	4.5		2.5-4.5
Alanine aminotransferase (U/L)	10	11		7-45
Aspartate aminotransferase (U/L)	11	11		8-43
Alkaline phosphatase (U/L)	46	45		35-104
Glucose (mg/DL)	116	96		70-140
Thyroid-stimulating hormone (mlU/L)		1.5		0.3-4.2
Creatine kinase (U/L)	50	51		26-192
C-reactive protein (mg/dL)		<3.0		< 8.0
Thiamine (nmol/L)			106	70-180
Antinuclear antibody (U)			0.3	< 1.0 (Negative)
Cyclic citrullinated peptide antibody (U)			< 15.6	< 20.0 (Negative)
Rheumatoid factor (IU/mL)			< 15	<15
Urinalysis				
Glucose		Negative		Negative
Ketones		Negative		Negative
Bilirubin		Negative		Negative
Protein		Negative		Negative
Leukocyte Esterase		Trace		Negative
Nitrite		Negative		Negative
Specific Gravity		1.012		1.002-1.030
pH		7.0		5.0-8.0
Red blood cells (urine RBC)		Moderate		0-2/hpf
White blood cells (urine RBC)		0/hpf		0-5/hpf

Due to recurrence of rhabdomyolysis symptoms and early laboratory findings of myoglobinuria, semaglutide discontinuation was again advised. The patient's symptoms promptly resolved following semaglutide discontinuation.

## Discussion

Rhabdomyolysis can present with multiple symptoms related to muscle injury including muscle pain, weakness, and dark urine. The diagnosis comprised clinical symptoms and elevated CK levels. Severe cases of rhabdomyolysis can present with complications including electrolyte abnormalities, cardiac arrhythmias, and acute renal failure [1,2]. Prior studies have associated a higher CK level with increased disease severity and an incidence of acute renal failure of 10-30% in rhabdomyolysis patients [5,6]. Myoglobinuria is caused by excessive cell death due to damage to the cell membrane of myocytes, resulting in the release of myoglobin into the bloodstream. Following this, intracellular components such as CK are released [7]. Given myoglobin has a short half-life of two to three hours, the amount detected in the urine can peak and normalize within 24 hours of muscle injury [8]. The causes of rhabdomyolysis vary, and several medications and substances have been noted to increase the risk of this condition [9-11]. 

Research on GLP-1 agonists continues to grow and evidence shows these medications are very effective in improving weight loss, managing glucose levels, and improving cardiorenal function [12]. GLP-1 agonists’ mechanism of action includes binding to GLP-1 receptors that are primarily found in the pancreas, but they can also be found in other organs. Specifically, it has been noted that GLP-1 agonists can increase glucose uptake and vascular perfusion within skeletal muscles [12]. Given the effect GLP-1 agonists can have on skeletal muscles, we hypothesize that there is a correlation between these medications and muscular injury that is yet to be elucidated. Notably, our patient tolerated liraglutide but experienced this adverse event with semaglutide; thus, it remains unclear if rhabdomyolysis is potentially a class effect of GLP-1 agonists or is limited to semaglutide specifically. 

In our patient, discontinuation of semaglutide resulted in vast improvement of her symptoms on two separate occasions. Evaluation of her symptoms suggested rhabdomyolysis, initially due to elevated CK, and then on retrial due to myoglobinuria. Extensive evaluation for other potential causes was negative. Awareness of this association is of utmost importance for clinicians, as reports linking GLP-1 agonists to rhabdomyolysis have not been previously described in the literature [13].

## Conclusions

In patients presenting with symptoms and laboratory evidence of rhabdomyolysis, an association between semaglutide and the presenting symptoms should be considered. It is important for physicians to recognize this potential adverse event of semaglutide and to promptly discontinue the drug to avoid potentially severe complications associated with rhabdomyolysis. Additional studies detailing the mechanism by which semaglutide, and potentially the class of GLP-1 agonists, results in rhabdomyolysis are warranted.
